# Could the IL‐17 cytokine family be a promising target for the treatment of palmoplantar pustulosis?

**DOI:** 10.1111/jdv.70393

**Published:** 2026-04-25

**Authors:** Ulrich Mrowietz, Sascha Gerdes

**Affiliations:** ^1^ Department of Dermatology, Center for Inflammatory Skin Diseases University Medical Center Schleswig‐Holstein Campus Kiel Germany

Palmoplantar pustulosis (PPP) is a rare, chronic inflammatory skin disease whose lesions are confined to the palms and/or soles. The aetiology of the disease is unknown, and differences in prevalence and clinical characteristics exist between Asian and Caucasian populations. PPP causes significant impairment in the use of the hands and/or feet. One hallmark of PPP is its resistance to treatment, and no gold standard for PPP treatment has yet been defined.

Several scientific studies suggest a link between inflammation/pustulation and interleukin‐17 (IL‐17) cytokines, such as IL‐17A and IL‐17F. Notably, IL‐17F has recently emerged as a dominant player in PPP.^1^ However, many other pathways have been implicated in PPP's pathology, resulting in a heterogeneous picture. This complexity is further increased by PPP occurring during biologic therapy, particularly TNF‐inhibiton. This ‘paradoxical’ PPP has also been observed in patients treated with IL‐17 inhibitors. Clinically, genuine and ‘paradoxical’ PPP are similar.

Case reports, case series and randomized controlled trials (RCTs) help to delineate potential targets associated with robust treatment responses. In the 2PRECISE study, secukinumab (an anti‐IL‐17A antibody) showed limited efficacy in PPP at the primary endpoint at week 16 (PPPASI75 of 26.6%), increasing to 41.8% at week 52 (open‐label extension) when administered at a dose of 300 mg in predominantly Caucasian patients.^2^ Brodalumab is a therapeutic antibody that blocks the IL‐17 receptor, thereby inhibiting signalling of IL‐17A and IL‐17F as well as their homo‐ and heterodimers. In Japanese PPP patients, brodalumab achieved a PPPASI75 of 36% after 16 weeks of treatment, compared to 8.1% in the placebo group. In the study by Murakami et al., the data from an open‐label extension of the brodalumab trial up to 68 weeks are reported.^3^ Among patients who received brodalumab continuously, 74.3% achieved a PPPASI75 response. Brodalumab has meanwhile been approved for the treatment of PPP in Japan. Consequently, the IL‐23p19‐driven inflammatory pathway has been explored as a therapeutic principle for PPP, with guselkumab showing limited efficacy in RCTs.

Recently, case series have shown that systemic janus kinase inhibitors (JAKi), such as upadacitinib, are highly effective in treating PPP; however, RCTs are still lacking. JAK inhibitors act as immunosuppressants by blocking the generation of multiple inflammatory mediators. The well‐established immunosuppressant drug ciclosporin is also highly effective in treating PPP, but its use is limited due to the potential for adverse events. Another interesting approach is the use of topical JAKi such as delgocitinib or ruxolitinib, and initial case reports are promising. Apremilast, a phosphodiesterase 4 inhibitor, has also been shown to be effective in the treatment of PPP.

Mainly in Japan, there is a particular emphasis on holistic PPP management that takes risk and triggering factors into account, as well as drug treatment. Tobacco smoking is the most important risk factor, with smoking cessation leading to an improvement in symptoms. Focal infections (e.g. tonsillitis and periodontitis) can induce or exacerbate PPP, and the respective treatments can provide long‐lasting benefit. Periodontitis is closely linked to smoking and shows an IL‐17 inflammatory signature with IL‐17F predominance.^4^ This may explain why blocking the IL‐17 receptor (brodalumab) or using bispecific anti‐IL‐17A/F antibodies (bimekizumab) may be more effective in PPP than IL‐17A inhibitors. Consequently, a novel bispecific nanobody (sonelokimab) is currently being tested in RCTs for PPP.

As the pathophysiology of PPP (including ‘paradoxical’ PPP) remains unclear, treatment is largely empirical, with arguments supporting both broader immunosuppression (ciclosporin, JAK inhibitors) and targeted therapy (IL‐17, IL‐23p19 inhibitors). In any case, drug treatment must be complemented by risk/trigger factor management to achieve sustained treatment outcomes.

## FUNDING INFORMATION

No funding was received for this article.

## CONFLICT OF INTEREST STATEMENT

Ulrich Mrowietz has been an advisor and/or received speakers' honoraria of the following companies: AbbVie, Aditxt, Almirall, Amgen, Biogen, Boehringer‐Ingelheim, Bristol‐Myers Squibb, Eli Lilly, Immunic, Janssen‐Cilag, LEO Pharma, Merck, Sharp & Dohme, Novartis, Phi‐Stone, SelectION, UCB Pharma, UNION therapeutics. Sascha Gerdes has been an advisor and/or received speakers' honoraria and/or received grants and/or participated in clinical trials of the following companies: AbbVie, Acelyrin Inc., Adimune, Inc., Affibody AB, Akari Therapeutics Plc, Almirall‐Hermal, Alumis Inc., Amgen, Apogee Therapeutics, Argenx BV, Aristea Therapeutics, Astra Zeneca, Bristol‐Myers Squibb, Boehringer‐Ingelheim, Celgene, Dermira, Eli Lilly, Galderma, Hexal AG, Incyte Inc., Janssen‐Cilag, Johnson & Johnson, Klinge Pharma, Kymab, Leo Pharma, Medac, MoonLake Immunotherapeutics AG, MSD, Neubourg Skin Care GmbH, Novartis, Pfizer, Pierre Fabre, Principia Biopharma, Regeneron Pharmaceutical, Sandoz Biopharmaceuticals, Sanofi‐Aventis, Santis, UCB Pharma.

## Data Availability

Data sharing is not applicable to this article as no datasets were generated or analysed during the current study.
